# Dichloroacetate blocks aerobic glycolytic adaptation to attenuated measles virus and promotes viral replication leading to enhanced oncolysis in glioblastoma

**DOI:** 10.18632/oncotarget.2838

**Published:** 2014-12-02

**Authors:** Chunyan Li, Gang Meng, Lei Su, Aiping Chen, Mao Xia, Chun Xu, Decai Yu, Aiqin Jiang, Jiwu Wei

**Affiliations:** ^1^ Jiangsu Key Laboratory of Molecular Medicine, Medical School and the State Key Laboratory of Pharmaceutical Biotechnology, Nanjing University, China; ^2^ Nanjing University Hightech Institute at Suzhou, Suzhou, China; ^3^ Drum Tower Hospital, Medical School of Nanjing University, Nanjing, China; ^4^ Zhongda Hospital, Medical School of Southeast University, Nanjing, China

**Keywords:** measles virus, glycolysis, dichloroacetate, glioma, oncolysis

## Abstract

Targeting reprogrammed energy metabolism such as aerobic glycolysis is a potential strategy for cancer treatment. However, tumors exhibiting low-rate glycolysis or metabolic heterogeneity might be resistant to such treatment. We hypothesized that a therapeutic modality that drove cancer cells to high-rate glycolysis might sensitize cancer cells to interference directed against metabolic flux. In this study, we found that attenuated oncolytic measles virus Edmonston strain (MV-Edm) caused glioblastoma cells to shift to high-rate aerobic glycolysis; this adaptation was blocked by dichloroacetate (DCA), an inhibitor of glycolysis, leading to profound cell death of cancer cells but not of normal cells. DCA enhanced viral replication by mitigating mitochondrial antiviral signaling protein (MAVS)-mediated innate immune responses. In a subcutaneous glioblastoma (GBM) xenograft mouse model, low-dose MV-Edm and DCA significantly inhibited tumor growth in vivo. We found that DCA impaired glycolysis (blocking bioenergetic generation) and enhanced viral replication (increasing bioenergetic consumption), which, in combination, accelerated bioenergetic exhaustion leading to necrotic cell death. Taken together, oncolytic MV-Edm sensitized cancer cells to DCA, and in parallel, DCA promoted viral replication, thus, improving oncolysis. This novel therapeutic approach should be readily incorporated into clinical trials.

## INTRODUCTION

Metabolic reprogramming has emerged as a key hallmark of cancer [1]. To provide sufficient bioenergetic and biosynthetic intermediates to support rapid cell growth and proliferation, cancer cells alter their metabolic flux distinct from the surrounding tissue. A well-known phenomenon observed in most cancer cells is a shift, regardless of oxygen supply, to aerobic glycolysis, termed “Warburg effect”, in which pyruvate is directly converted to lactic acid instead of entering the citric acid (TCA) cycle [2, 3]. Efforts have been made to target reprogrammed metabolism alone or in combination with cancer chemotherapy both in preclinical and clinical studies [3].

The orphan drug dichloroacetate (DCA) is a pyruvate mimetic. DCA has been used to treat human hereditary mitochondrial metabolic diseases and lactic acidosis for more than 30 years [4, 5]. It specifically inhibits the kinase family member pyruvate dehydrogenase kinase (PDK) leading to reactivation of pyruvate dehydrogenase (PDH), a key enzyme that shifts the flux of pyruvate into mitochondria to promote glucose oxidation instead of glycolysis [6]. DCA has been recently evaluated in several pre-clinical cancer therapies including prostate [7], endometrial [8], colon [9, 10], breast cancer [11], lung cancer [12], and in a clinical study for glioblastoma [13]. In the latter study, DCA was shown to depolarize mitochondria, increase mitochondrial reactive oxygen species, and induce apoptosis in glioblastoma (GBM) cells, as well as in putative GBM stem cells. A recent study shows that the antitumor activity of DCA is correlated with glycolytic bias [14]. It is speculated that DCA holds promise against most cancers with high glycolysis-dependency.

However, cancer cells within the same tumor are intrinsically heterogeneous due to variable blood supply, oxygenation status, tissue pH, etc [15]. Metabolic bias has also been identified in glioblastoma [16-18]. Metabolic heterogeneity within a tumor mass, i.e., cells exhibiting differential glycolysis-dependency, may contribute to variable responses to therapies directed against glycolysis. For instance, glioma cells with a glycolysis-dependent phenotype displayed low tolerance to glucose starvation, whereas glioma cells with an oxidative phosphorylation-dependent phenotype exhibited prolonged survival under glucose starvation [17].

Viral replication is dependent on the host cellular metabolism for biomaterials and bioenergetics. As well, viral infection alters cellular metabolism to facilitate viral reproduction [19, 20]. An increased rate of glycolysis has been observed in cells following infection by any number of viruses including influenza virus [21], feline leukemia virus [22], Rous sarcoma virus [23], Avian Sarcoma Viruses [24], Rubella-virus [25], cytomegalovirus [26, 27], Mayaro virus [28], Newcastle disease virus [29], and poliomyelitis virus [30]. This raises the possibility that oncolytic viruses could be employed to propel or synchronize cellular metabolism of cancer cells to high-rate glycolysis.

Replicating oncolytic viruses are emerging as a promising modality for the treatment of malignant gliomas and other malignancies. Attenuated measles virus Edmonston strain (MV-Edm) has exhibited potent oncolytic activity in some preclinical studies against human lymphoma [31], multiple myeloma [32], ovarian cancer [33, 34], malignant glioma [35, 36], and ﬁbrosarcoma [37]. Due to its oncolytic efficacy and excellent safety record, this oncolytic measles virus has been evaluated for treatment of cutaneous T-cell lymphoma [38], and is now being tested in several phase I/II clinical studies [39].

The role of MV-Edm in altering host metabolism is unclear. Given that cancers with high dependency on glycolysis are more sensitive to DCA, we hypothesized that a therapeutic modality employing an oncolytic virus to drive cancer cells to a high glycolysis-dependent phenotype might sensitize the tumor to DCA. In this study, we have investigated the metabolic adaptation of GBM cells to MV-Edm infection and the consequence of subsequent DCA treatment *in vitro* and in a mouse xenograft GBM tumor model. We found an improved antitumor effect at a relatively low infectious dose of virus in combination with DCA.

## RESULTS

### MV-Edm infection shifts cellular metabolism to a high-rate glycolytic adaptation in glioma cells

**Figure 1:**
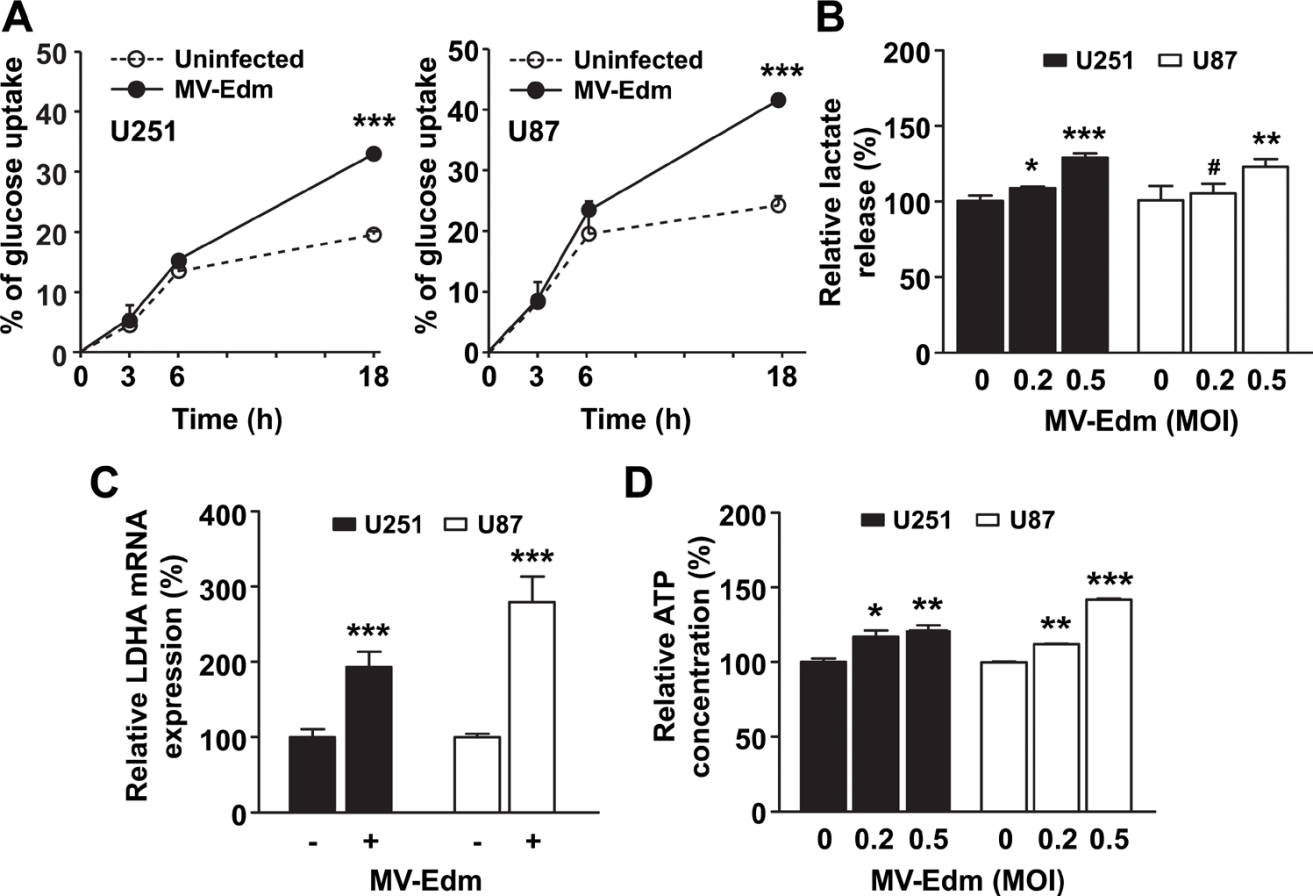
MV-Edm shifts cellular metabolism to a high-rate glycolytic adaptation (A) U251 and U87 GBM cells were infected with or without MV-Edm (MOI = 0.2) as indicated. Supernatant was harvested at 0, 3, 6, and 18 h after infection, and glucose concentration was determined. Glucose uptake was determined as the percent reduction in glucose concentration at each time point compared to initial (0 h) glucose concentration in the medium. (B) U251 and U87 cells were infected with MV-Edm at an MOI of 0.2 or 0.5, or left untreated. Supernatant was harvested 6 h later, and lactate release was determined. (C) *LDHA* expression was quantified by qRT-PCR using mRNA harvested from U251 and U87 cells infected with or without MV-Edm (MOI = 0.2) for 6 h. (D) ATP content was determined in cell lysates from U251 and U87 cells infected for 6 h with MV-Edm at a MOI of 0, 0.2, or 0.5. Data are Mean + SD of triplicates. Similar results were obtained in three independent experiments. * p < 0.05, ** p < 0.01, *** p < 0.001, # p > 0.05.

As little is known about the metabolic alterations to MV-Edm infection in cancer cells, we first determined the glycolytic adaptation to viral infection in glioma cell lines U251 and U87. We found that glucose uptake in MV-Edm infected cells was rapidly upregulated (6 h post-infection), and increased 15% to 20% (18 h post-infection) compared to uninfected GBM cells (Figure 1A). The increased glucose uptake following MV-Edm infection could be contributed by either increased aerobic glycolysis or glucose oxidation by TCA cycles in mitochondria. To discriminate between these possibilities, we monitored the generation of lactate, a product normally generated from pyruvate under hypoxic conditions, but when it occurs under normoxic conditions is known as aerobic glycolysis. We found that lactate release was rapidly increased in cancer cells even at 6 h after MV-Edm infection under normoxia (Figure 1B). Consistently, the expression of LDHA mRNA, which encodes a key enzyme that converts pyruvate to lactate, was significantly upregulated in MV-Edm infected GBM cells (Figure 1C). Correspondingly, ATP generation in MV-Edm infected cells was transiently increased at early time points, e.g., 6 h post-infection (Figure 1D), indicating that cells entered into high-rate energy generation. Together, these results suggest that MV-Edm infection shifted cellular metabolism to high-rate aerobic glycolysis.

### DCA blocks glycolytic adaptation to MV-Edm in GBM cells

**Figure 2:**
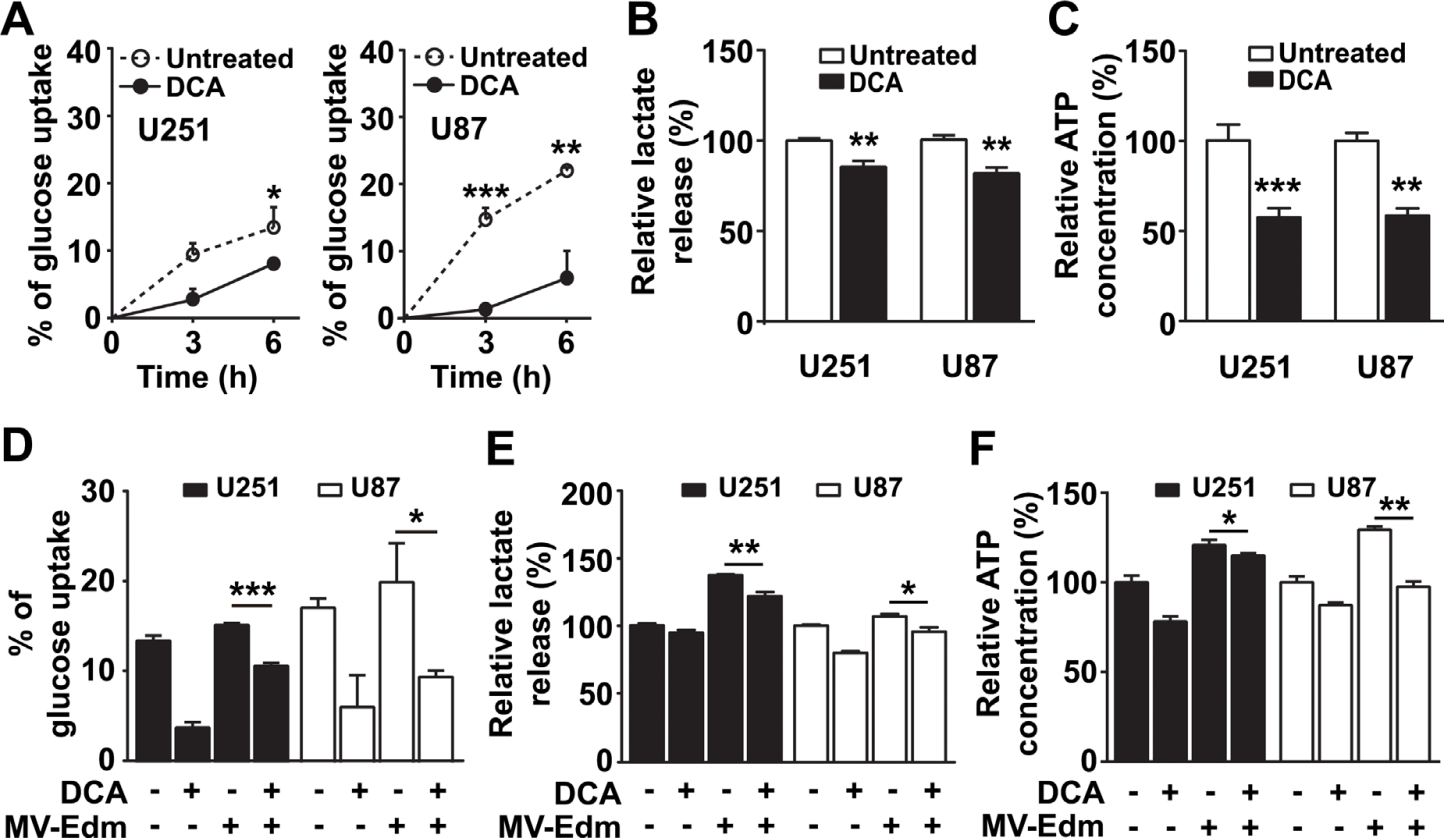
DCA blocks MV-Edm-induced glycolysis (A) Glucose content was determined in the supernatant harvested from U251 and U87 GBM cells treated with DCA (5 mM) for 0, 3 and 6 h. Glucose uptake was reported as the percent reduction in glucose concentration at each time point compared to the initial (0 h) glucose level. (B & C) U251 and U87 cells were treated with or without DCA (5 mM) for 12 h; then (B) supernatant was tested for lactate production and (C) cell lysates were tested for ATP generation. (D - F) U251 and U87 cells were treated for 6 h with DCA (5 mM), MV-Edm (MOI = 0.2), MV-Edm combined with DCA, or left untreated; supernatant was then tested for (D) glucose uptake and (E) lactate release; cell lysates was detected for (F) ATP content. Means + SD of triplicate cultures are shown. Similar results were obtained in three independent experiments. * p < 0.05, ** p < 0.01, *** p < 0.001.

Previous studies have confirmed that DCA inhibits the conversion of pyruvate to lactate. We wanted to determine if DCA blocked MV-Edm induced high-rate aerobic glycolysis. We first confirmed that DCA efficiently inhibited aerobic glycolysis in GBM cells, which was evidenced by decreased glucose uptake (Figure 2A), reduced lactate production (Figure 2B), and reduced ATP generation (Figure 2C) under normoxia. We further found that glucose uptake (Figure 2D) and lactate production (Figure 2E) and ATP generation (Figure 2F) were significantly decreased in MV-Edm/DCA treated cells compared to MV-Edm infection alone. These results show that DCA efficiently blocked glycolytic adaptation to MV-Edm infection in GBM cells.

### DCA promotes MV-Edm replication by impairing MAVS-mediated anti-viral innate immune responses

**Figure 3:**
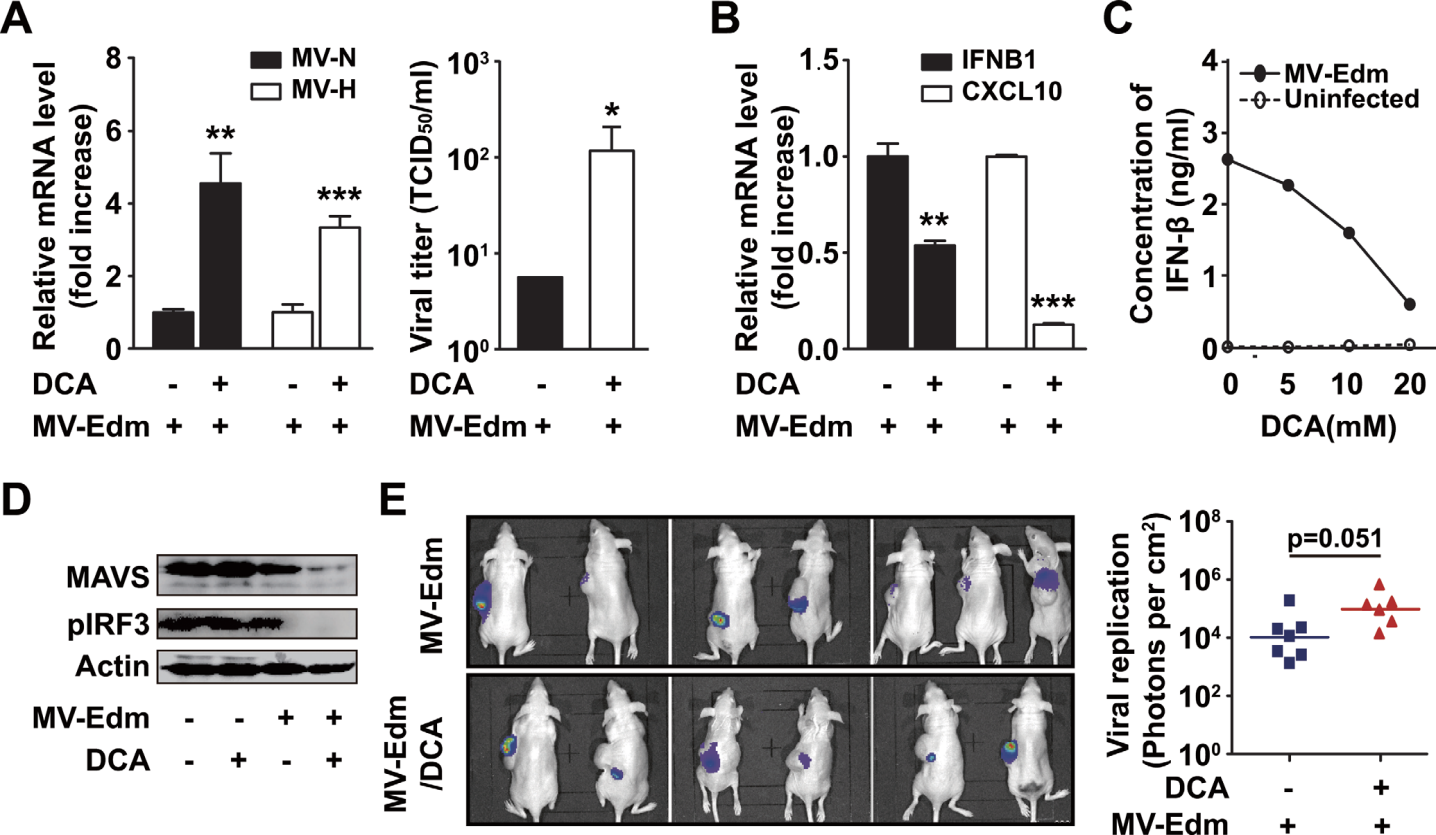
DCA promotes viral replication by disrupting MAVS-mediated anti-viral immune responses (A) U251 cells were infected with MV-Edm (MOI = 0.2) in the presence or absence of DCA (5 mM) for 24 h, then the total RNA in cells was harvested for the determination of viral genes encoding H and N proteins by qRT-PCR (left panel), or the supernatant was harvested for determination of TCID_50_ on Vero cells (right panel). Similar results were obtained in two independent experiments. (B) U251 cells were infected with MV-Edm (MOI = 0.2) in the presence or absence of DCA (5 mM) for 24 h, then the expression of *IFNB1* and *CXCL10* mRNA was determined by qRT-PCR. Means + SD of triplicates are shown. Similar results were obtained in three independent experiments. (C) U251 cells were infected with MV-Edm (MOI = 0.2) in the absence or presence of DCA (5, 10, or 20 mM) for 12 h, and supernatants were then harvested, and the protein level of IFN-β was measured by ELISA. (D) U251 cells were treated with DCA (5 mM), MV-Edm (MOI = 0.2), MV-Edm combined with DCA, or left untreated, and cultured for 24 h. Cell lysates were then harvested for immunoblotting against MAVS or pIRF3; β-actin was used as a loading control. A representative result from two independent experiments is shown. (E) U87 cells were inoculated subcutaneously into Balb/c nude mice. When tumors reached a palpable size, one group of mice received DCA (70 mg/L) in the drinking water for 10 d (n = 6). Another group was left untreated (n = 7). Then both groups of mice were injected with MV-Edm-Luc (4 × 10^5^ pfu per mouse) via tail vein. Luciferase activity was monitored by an in vivo luminescence imaging system 72 h after virus injection (left panel). Photons per cm^2^ tumor were quantified (right panel). * p < 0.05, ** p < 0.01, *** p < 0.001.

Effective viral replication within cancer cells is crucial for oncolysis. Having shown that DCA blocked glycolytic adaptation to MV-Edm infection, we wondered if this effect compromised viral replication. To our surprise, in the presence of DCA viral replication was increased 3 to 4 fold in U251 cells, as determined by expression of viral structural H- and N-protein genes 24 h post-infection (Figure 3A left panel). Consistently, we found that the viral particles in the supernatant were also increased (Figure 3A right panel). As type-I interferons play key roles in controlling viral replication, we evaluated the expression of IFNB1/IFN-β and CXCL10/IP-10, an interferon inducible protein. We found that mRNA levels of both IFNB1 and CXCL10 were significantly decreased in MV-Edm/DCA treated GBM cells (Figure 3B). We further confirmed that the decrease in IFNB1 mRNA expression correlated with decreased protein levels as determined by ELISA. IFN-β production was decreased in a dose-dependent manner after MV-Edm infection of DCA treated cells (Figure 3C). Moreover, we found that mitochondrial antiviral signaling protein (MAVS), a key adaptor protein in signaling during the anti-viral innate immune response, and its downstream target, phosphorylated IRF3, were dramatically decreased in GBM cells treated with MV-Edm/DCA (Figure 3D). We next evaluated viral replication in a GBM xenograft tumor model. Viral replication was monitored by *in vivo* imaging following intravenous injection of a genetically modified MV-Edm expressing a luciferase gene (MV-Edm-Luc) in U87 glioma-bearing mice. The mean luciferase activity in tumors, reflecting viral replication, was higher in mice treated with MV-Edm/DCA than in mice treated with MV-Edm alone (Figure 3E). Although the difference did not reach statistical significance between the two groups (p = 0.051), a trend of improved viral replication *in vivo* was evident. Taken together, the data suggest that DCA promotes MV-Edm replication by disrupting MAVS-mediated anti-viral immune responses.

### Combining DCA with low-dose MV-Edm enhances antitumor efficacy in GBM

**Figure 4:**
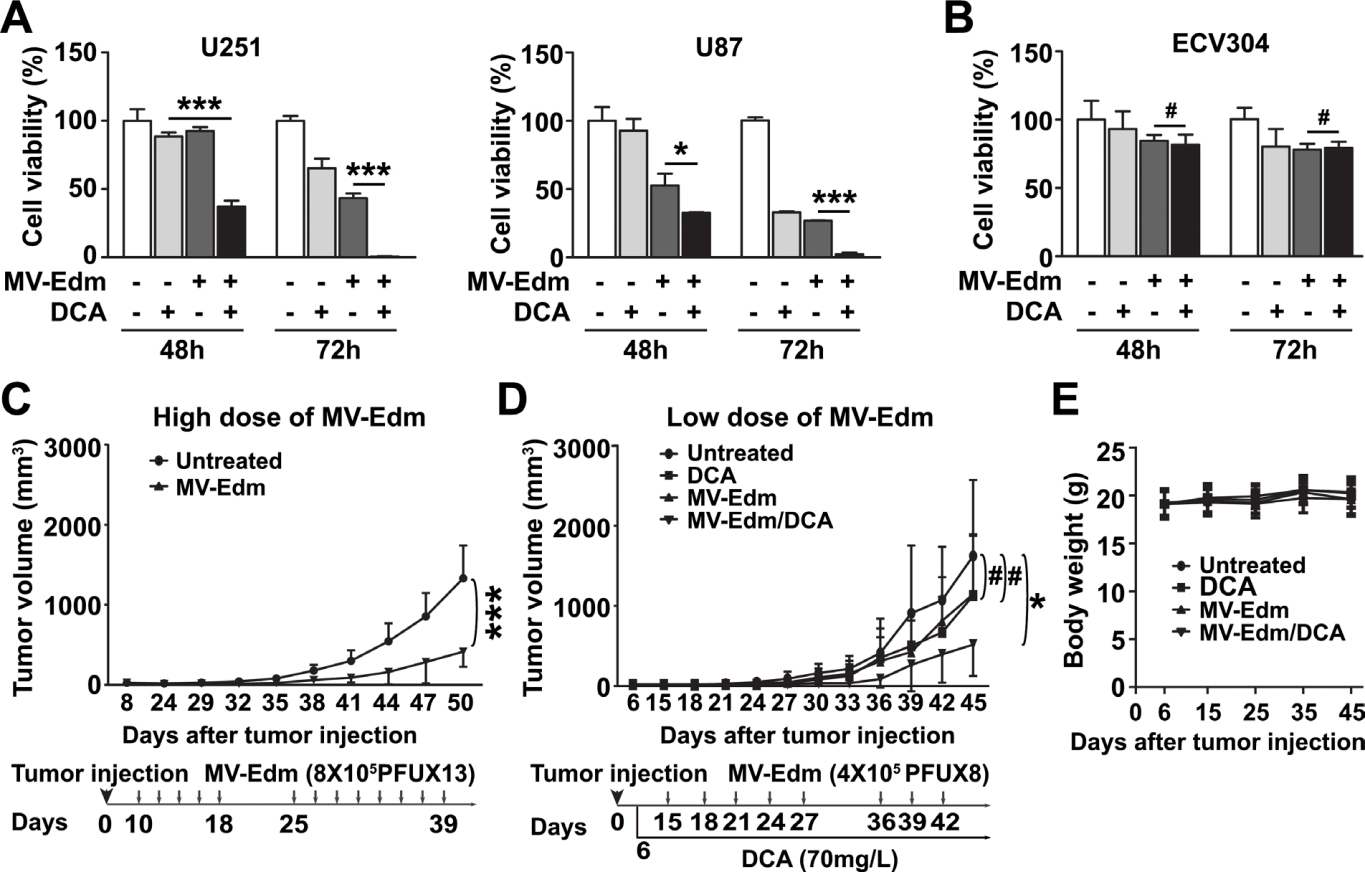
DCA combined with low-dose MV-Edm exerts an enhanced anti-tumor effect (A) U251 and U87 glioma cells or (B) ECV304 human endothelial cells were infected or uninfected with MV-Edm (MOI = 0.2) followed by addition of DCA at a concentration of 5 mM for 48 or 72 h. Untreated cells were used as negative controls. Cell viability was determined by trypan blue exclusion. Means ± SD of triplicates are shown. Similar results were obtained in three independent experiments. (C) Male Balb/c nude mice (6 to 8 week old) were injected subcutaneously with U87 cells in the left flank on day 0 and randomized to two groups (n = 8 per group). Tumors became palpable on day 5. Starting on day 10 after tumor inoculation, one group of mice received MV-Edm (8 × 10^5^ PFU per mouse) via tail vein injection every other day from day 10 to 18 and from day 25 to 39 (total dose of 1 × 10^7^ PFU MV-Edm). (D) Male Balb/c nude mice (6 to 8 week old) were injected subcutaneously with U87 cells in the left flank on day 0 and randomized to four groups as depicted. The treatment groups received DCA supplemented in drinking water (70 mg/L) on day 6 (n = 6), or received low-dose MV-Edm (4 × 10^5^ PFU per mouse) injection via tail vein every 3 days from day 15 to 27 and from day 36 to 42 (n = 6) (total dose of 3.2 × 10^6^ PFU), or received both DCA and MV-Edm administration (n = 6). An untreated group (n = 5) was used as a control. Data are mean ± SD. * p < 0.05, ** p < 0.01, *** p < 0.001, # p > 0.05. (E) Body weight of mice was monitored during the treatment. Means ± SD of each group at various time points are shown.

Having shown that DCA blocks aerobic glycolytic adaptation to MV-Edm, and that DCA promotes viral replication, we next investigated the antitumor activity of MV-Edm/DCA in GBM. *In vitro*, enhanced antitumor effects were achieved by combining low-dose MV-Edm (MOI = 0.2) with DCA at a concentration of 5 mM (Figure 4A). Importantly, we found that MV-Edm/DCA treatment had only minimal effects on the viability of the normal human endothelial cell line ECV304 (Figure 4B). Next, we wanted to know if low-dose MV-Edm combined with DCA could contribute to an improved therapeutic outcome *in vivo*. We established a GBM xenograft model by subcutaneous inoculation of U87 cells into Balb/c nude mice. First, we confirmed that MV-Edm infection produced a significant inhibition of tumor growth (Figure 4C). Then, employing a lower infectious dose of MV-Edm (total dose, 3.2 × 10^6^ PFU per mouse) we found that DCA combined with low-dose MV-Edm significantly inhibited tumor growth, whereas only marginal tumor inhibition was observed in mice receiving either DCA or low-dose MV-Edm single treatment (Figure 4D). The dosage of DCA was similar to previous studies [6, 12] and to that used clinically [13]. No significant side-effect was observed as evidenced by the comparable body weight among four groups (Figure 4E). These data show that DCA with low-dose MV-Edm improves therapeutic outcome.

### Accelerated exhaustion of bioenergetics leads to necrosis in GBM cells

**Figure 5:**
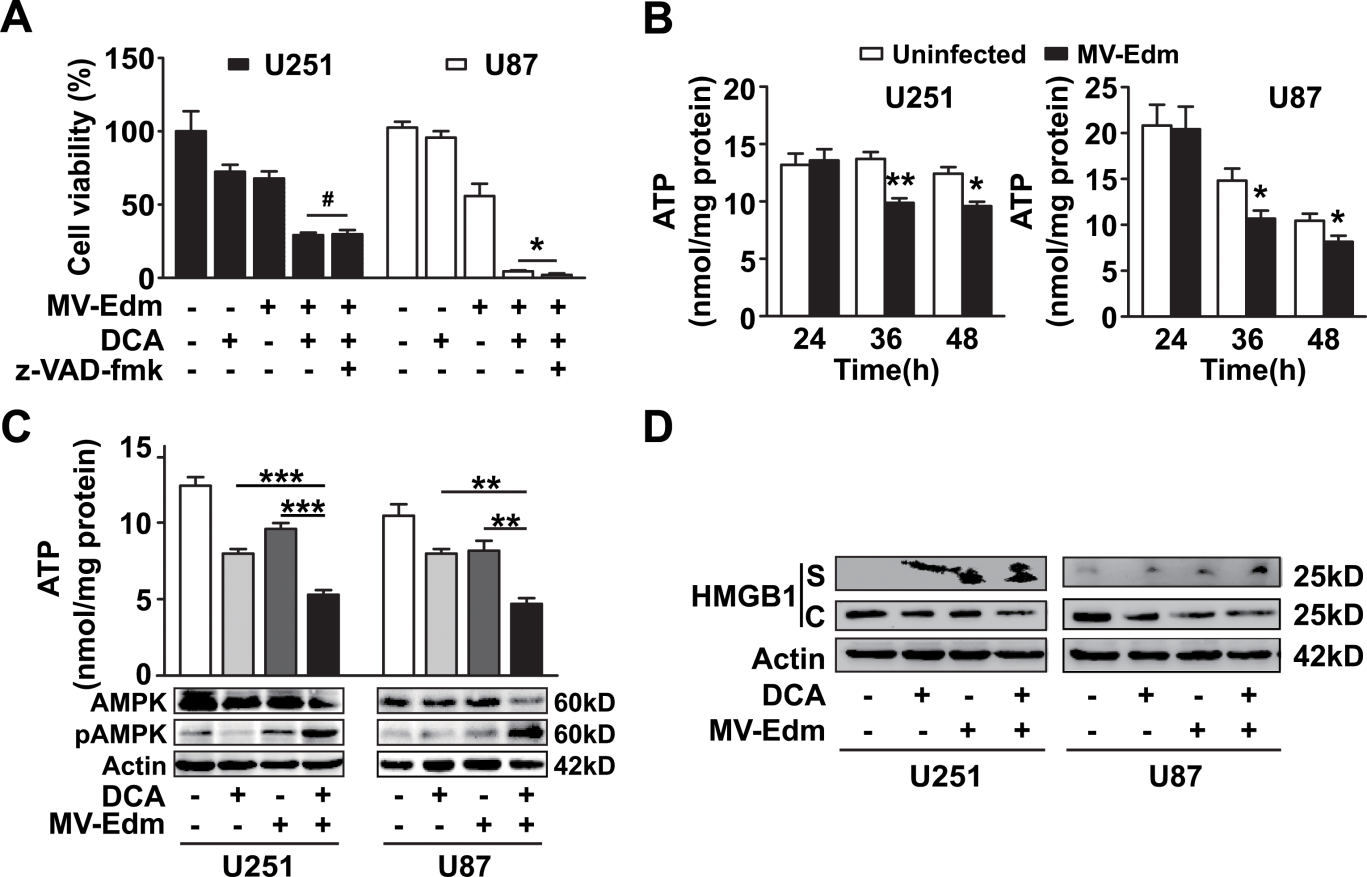
Necrosis contributes to MV-Edm/DCA mediated oncolysis by accelerated bioenergetics exhaustion (A) U251 and U87 cells were treated with DCA (5 mM), MV-Edm (MOI = 0.2), MV-Edm combined with DCA in the presence or absence of z-VAD-fmk (80 μM), or left untreated. Cell viability was determined by trypan blue exclusion 60 h post-treatment. Similar results were obtained in two independent experiments. (B) ATP content was determined in cell lysates harvested from U251 and U87 cells infected with MV-Edm at an MOI of 0.2 for 24, 36, or 48 h. Untreated cells were used as a negative control. Means + SD of triplicates are shown. Similar results were obtained in three independent experiments. (C) U251 and U87 cells were treated with DCA (5 mM), MV-Edm (MOI = 0.2), MV-Edm combined with DCA, or left untreated for 48 h. Cell lysates were then harvested for determination of ATP content (upper panel), or for immunoblotting against AMPK and phosphorylated AMPK (lower panel). Similar results were obtained in three independent experiments. (D) U251 and U87 cells were treated with MV-Edm (MOI = 0.2), DCA (5 mM), MV-Edm combined with DCA, or left untreated for 48 h. Cell lysates (C) and supernatants (S) were harvested for immunoblotting against HMGB1. β-actin was used as a loading control. Similar results were obtained in three independent experiments. * p < 0.05, ** p < 0.01, *** p < 0.001, # p > 0.05.

Finally, we sought to clarify the mechanism underlying enhanced antitumor activity of MV-Edm/DCA. Since apoptosis has been suggested as the mechanism of cell death induced by DCA or MV-Edm in glioma [13, 37], we investigated the contribution of apoptosis to the combined antitumor effect. We found that z-VAD-fmk, a pan caspase inhibitor, failed to inhibit MV-Edm/DCA induced cell death (Figure 5A). Given that DCA blocked glycolytic adaptation to MV-Edm (impairment of rapid energy generation) and that DCA promoted viral replication (promotion of energy consumption), we speculated that MV-Edm/DCA treatment might accelerate bioenergetic exhaustion. Having shown that ATP generation was transiently increased due to high-rate glycolytic adaptation upon MV-Edm infection (Figure 1D), we assessed cellular ATP levels at later timepoints. We found that ATP levels were significantly decreased along with viral replication 36 and 48 h after MV-Edm infection (Figure 5B). We found that ATP levels were dramatically decreased in MV-Edm/DCA treated GBM cells compared to cells with single treatment (Figure 5C). An energy crisis in cells treated with MV-Edm/DCA was further indicated by a massive increase in the level of pAMPK (Figure 5C), an energy sensor triggered by insufficient ATP supply. These data suggest that an accelerated bioenergetic shortage may dominantly contribute to the enhanced antitumor effect. In view of the fact that a cellular ATP shortage mainly leads to necrosis, and the fact that the high mobility group box1 (HMGB1) has been identified as a danger signal released from necrotic cells that can be used as a necrotic marker [40], we evaluated the HMGB1 level in the cytoplasm and supernatant of MV-Edm/DCA treated cells. We found that the HMGB1 level was increased in the supernatant and in parallel was decreased in the cytoplasm of MV-Edm/DCA treated GBM cells (Figure 5D), which confirmed necrotic cell death. These data imply that MV-Edm/DCA induces a severe bioenergetic crisis in GBM cells leading to necrotic cell death.

## DISCUSSION

We hypothesized that an intervention modality that initially drove cancer cells to high glycolysis-dependency followed by a treatment directed against glycolysis might improve the antitumor effect. In this study, we found that the self-replicating MV-Edm shifts cellular metabolism to a high-rate glycolytic adaptation, which can be efficiently targeted by DCA, leading to improved antitumor activity both *in vitro* and in a tumor-bearing mouse model. We did not observe any toxicity. Our data indicated that DCA promoted MV-Edm replication by impairing the MAVS-mediated anti-viral innate immune response. The therapeutic approach of combining DCA with low-dose MV-Edm produced an enhanced antitumor effect that resulted in dominant necrotic cell death due to a severe shortage of cellular ATP in GBM cells. Given that both MV-Edm and DCA have been effectively tested in clinical studies, this novel strategy could be readily moved from bench to bedside.

MV-Edm infection upregulates glycolysis under aerobic conditions (the Warburg effect) in glioblastoma cells, which was evidenced by increased glucose uptake, lactate production, and *LDHA* expression upon MV-Edm infection. We observed a rapid increase in ATP generation suggesting that cancer cells were shifted to a high-rate glycolytic adaptation. Similar to other viruses that upregulate glycolysis [33-42], the metabolic shift toward glycolysis presumably favors both viral replication and host cell survival. It is thought that viruses mobilize glycolysis of infected cells to provide sufficient nutrients and energy for viral replication, and infected cells shift to high-rate glycolysis for rapid generation of ATP to compensate for the “robbed bioenergetics”.

We found that DCA significantly improved oncolysis of low-dose MV-Edm both *in vitro* and *in vivo*. The improved antitumor effect was achieved through two distinct effects. First, DCA successfully blocked glycolytic adaptation to MV-Edm infection. Given that the antitumor activity of DCA is likely to be enhanced in cancer cells with more glycolysis-dependency [14], the conversion to high-rate glycolysis mediated by MV-Edm infection would make GBM cells more vulnerable to DCA induced cell death. Second, DCA promoted viral replication in GBM, which would enhance its oncolytic effect. The enhanced viral replication in MV-Edm/DCA treated cells is likely the consequence of impaired innate immune responses mediated by MAVS, a key adaptor protein in antiviral signaling [41]. The MAVS protein level was markedly decreased in GBM cells, suggesting that MV-Edm/DCA treatment enhanced MAVS degradation. However, the precise mechanism is unknown in our setting and needs further study. It has been reported that mitochondrial membrane potential (Δψm) is required for MAVS-mediated anti-viral signaling [42], and that DCA reduces Δψm via remodeling mitochondria [43]. It is possible that reduced Δψm (data not shown) might partially contribute to impaired MAVS signaling. The enhanced viral replication induced by DCA makes this strategy attractive, as viral replication is crucial for improving MV-Edm-mediated oncolysis. Thus, the combinational modality of MV-Edm/DCA represents an efficient and practical strategy for clinical oncolytic virotherapy.

The enhanced cell death was a result of necrosis rather than apoptosis. In MV-Edm/DCA treated cells, cell viability could not be rescued by the pan caspase inhibitor z-VAD-fmk. Previous studies show that ATP exhaustion predominantly leads to necrosis [44]. We confirmed that the necrosis was a consequence of a severe bioenergetics crisis, as MV-Edm/DCA treatment dramatically decreased ATP generation. Necrotic cell death was directly confirmed by finding increased levels of HMGB1 in the supernatant of MV-Edm/DCA treated cells, which was accompanied by a parallel decrease in HMGB1 expression in the cytoplasm.

The bioenergetic exhaustion in MV-Edm/DCA treated GBM cells was the consequence of two synchronous factors: enhanced bioenergetics consumption and impaired bioenergetics generation. On the one hand, despite the rapid increase in ATP generation upon MV-Edm infection at early time points due to the cellular glycolytic adaptation, the ATP level was decreased along with viral replication 36 h post-infection due to massive bioenergetics consumption. Moreover, DCA promoted viral replication, and thus, accelerated ATP exhaustion in MV-Edm/DCA treated cells. On the other hand, DCA blocked aerobic glycolytic adaptation to MV-Edm infection, which further exacerbated the bioenergetics crisis. It is also plausible that the improved viral replication might facilitate viral spread to neighboring non-infected cells, which in turn, would sensitize more GBM cells to DCA mediated inhibition of glycolysis, a vicious cycle ultimately leading to enhanced antitumor activity.

It is important to note that no obvious side-effects were observed in treated mice. The safety of this strategy was also confirmed by our observation *in vitro* that normal human endothelial cells are resistant to MV-Edm/DCA treatment. Possible explanations might be that MV-Edm is non-toxic or mildly-toxic to normal cells and that under normoxic conditions normal cells shunt glycolytic pyruvate into the TCA cycle rather than conversion to lactate. These advantages may provide a wide therapeutic-window for a MV-Edm/DCA treatment modality.

Several oncolytic viruses have been shown to elicit anti-tumor immune responses [38, 45-47]. As well, DCA is able to enhance antitumor immunity [48]. Thus, it would be interesting to determine whether MV-Edm/DCA treatment elicits a more profound antitumor immunity. Because our *in vivo* study was conducted in immunocompromised mice bearing human GBM cells (as measles virus only infects primates or humans), immunologic reactions could not be reliably assessed. Further studies using immunocompetent mice, e.g., CD46 transgenic mice, may permit a comprehensive analysis. Of note, recent studies have shown that lactate may compromise anti-tumor immune responses [49, 50], and we showed that DCA significantly decreased lactate production in cancer cells infected with MV-Edm. Thus, MV-Edm/DCA modality might contribute to amplify the anti-tumor immune responses by reducing tumor lactate production, which deserves further intensive investigation. It has been shown recently that mTOR inhibitor rapamycin decreases lactate production in cancer cell lines regardless of normoxia or hypoxia [51, 52], which may also extend our further anticancer investigation to target aerobic glycolytic adaptation to oncolytic viruses by mTOR inhibitors.

In summary, our work provides a novel, clinically relevant, therapeutic strategy that provides a rationale for combining oncolytic virotherapy with a treatment modality targeting reprogrammed cancer cell metabolism. The strategy is based on two promising antitumor strategies, both of which have been tested in clinical studies, and which can be readily translated to clinical cancer therapy.

## METHODS

### Cells and reagents

The human glioblastoma cell lines U251 and U87 and human ECV304 endothelial cells and Vero cells were cultured in DMEM supplemented with 5% fetal bovine serum, 2 mM L-glutamine, 100 U/l penicillin and 0.1 mg/ml streptomycin (all from Invitrogen, Karlsruhe, Germany). Cultures were maintained in a humidified incubator with 5% CO_2_ at 37°C. Dichloroacetate (DCA), Adenosine Triphosphate (ATP), and z-VAD-fmk were all obtained from Sigma-Aldrich (Taufkirchen, Germany).

### Glucose uptake and lactate release assay

Supernatants were harvested from cultured U251 and U87 cells at various time points after treatment. To determine glucose uptake, glucose concentration in culture supernatants was determined using a glucose assay kit (Shanghai Rongsheng Biotech, Shanghai, China, F006) according to the manufacturer's instructions, and quantified by absorption at 450 nm. Lactate generation was measured using a lactate assay kit according to the manufacturer's instruction (Nanjing Jiancheng Bioengineering Institute, Nanjing, China, A019-2). Briefly, NAD^+^ is added to the media and is converted to NADH stoichiometrically by lactate in the medium. The levels of NADH were quantified colorimetrically at 530 nm.

### ATP detection

Intracellular ATP was measured by a luciferin-luciferase method using with an ATP assay kit (Beyotime Inst. Biotech, Jiangsu, China, S0026). In brief, cells were washed once with PBS and transferred to lysis buffer. The supernatants were harvested by centrifugation at 12000 g for 10 min at 4°C, and mixed with ATP detection buffer and analyzed by luminescence spectrometry. The final ATP content of each sample was normalized to protein concentration measured by BCA Protein Assay Kit (Beyotime Inst. Biotech, Jiangsu, China, P0012).

### Virus titration

MV-Edm and MV-Edm expressing a reporter gene luciferase (MV-Edm-luc, kindly provided by S. Russell, Mayo Clinic, MN, USA) were propagated in Vero cells following infection at an MOI of 0.02 in 2 ml OptiMEM (Invitrogen, 31985-062) at 37°C for 3 h. The medium was changed to DMEM supplemented with 2% FCS and cells were incubated at 37°C for 1 day before being transferred to 32°C for another day. Cells were harvested, and viral particles were released by two cycles of snap freezing in liquid nitrogen and thawing in 37°C water bath. Or the supernatant from glioma cells infected with MV-Edm in the presence or absence of DCA were harvested, centrifuged, and stored at −80°C until used. Viral titers were determined by 50% end-point dilution assays (TCID_50_) on Vero cells.

### Cell viability assay

Cells were harvested using trypsin/EDTA solution and stained with trypan blue. Viability was then determined by trypan blue exclusion using a Countstar Automated cell counter (Inno-Alliance Biotech Inc., Wilmington, USA).

### ELISA

Supernatants from treated or untreated cells were harvested, centrifuged, and stored at −80°C until used. Samples were analyzed using an ELISA assay for detection of IFNB1/IFN-ß (R&D Systems, Minneapolis, MN, 41410-1A) according to the manufacturer's protocol.

### Quantitative RT-PCR

For quantitative RT-PCR (qPCR), total cellular RNA was extracted with TRIZOL (Invitrogen, 15596-026) and 1 μg of RNA was reverse-transcribed using the Master Mix Perfect Real Time kit (TaKaRa, Shiga, Japan, DRR036A) according to the manufacturer's protocol. qPCR was performed using the Real-Time PCR system (ABI 7300, Advanced Biosystems, Foster, CA). Gene expression was calculated with the comparative Ct method and normalized to the endogenous levels of GAPDH. Primer sequences used for qPCR were: *CXCL10*, 5′-CTTCCAAGGATGGACCACACA-3′ and 5′-CCTTCCTACAGGAGTAGTAGCAG-3′; *IFNB1*, 5′-CTTGGATTCCTACAAAGAAGC-3′ and 5′-CATCTCATAGATGGTCAATGC-3′; *MV-Edm N-protein*, 5′-ACATTAGCATCTGAACTCGGTATCAC-3′ and 5′-TTTTCGCTTTGATCACCGTGTA-3′; *MV-Edm H-protein*, 5′-GATGACAAGTTGCGAATGGAGA-3′ and 5′-GACAAGACCCCGTATGAAGGAA-3′; *LDHA*, 5′-GCCCGACGTGCATTCCCGATTCCTT-3′ and 5′-GACGGCTTTCTCCCTCTTGCTGACG-3′; *GAPDH*, 5′-CCACCCATGGCAAATTCCAT-3′ and 5′-TCTAGACGGCAGGTCAGGTCC-3′.

### Western blot

Cells were lysed in RIPA buffer containing a protease inhibitor cocktail (Roche, Mannheim, Germany, 11873580001). Protein concentration was determined. Equal amounts of protein were separated by SDS-PAGE and electrophoretically transferred onto a PVDF membrane (Roche, 03010040001). After blocking with 5% nonfat milk in Tris-buffered saline containing 0.1% Tween-20, the membrane was incubated with specific primary antibodies, followed by incubation with appropriate horseradish peroxidase–conjugated secondary antibodies. Signals were developed using an enhanced chemiluminescence reagent (Millipore, Darmstadt, Germany, WBKLS0500) and captured on an Alpha Innotech Fluor Chem FC2 imaging system (Alpha Innotech, San Leanardo, CA). Antibodies used in this study were: rabbit anti-β-ACTIN (Biosynthesis Biotechnology, Beijing, China, bs0061R, 1:1000), rabbit anti-HMGB1 (Abcam, Hong Kong, China, ab191583, 1:1000), rabbit anti-AMPK/pAMPK (Cell Signaling Technology, Danvers, MA, #2532 / #2531, 1:1000), rabbit anti MAVS (Abcam, ab31334, 1:500) and HRP-conjugated secondary antibodies (Multisciences, Hangzhou, China, GAR007 and GAM007, 1:5000).

### Visualization of MV-Edm replication *in vivo*

Male Balb/c nude mice (6-8 week old) were injected subcutaneously with 1×10^6^ U87 cells in the left flanks and randomized to 2 groups. When tumors reached palpable size, one group of mice was treated for 10 days with DCA (70 mg/L in drinking water). Both groups of mice then received 4 × 10^5^ PFU MV-Edm-Luc via tail vein injection. Three days after MV-Edm injection, mice were anesthetized and injected intraperitoneally with D-luciferin (Gold Biotechnology, St. Louis, MO) and subjected to luciferase assay using the IVIS Lumina XR system (Caliper Life Sciences, Hopkinton, MA). The level of firefly luciferase was expressed as the ROI value normalized to tumor volume.

### *In vivo* treatment with high-dose MV-Edm

Male Balb/c nude mice (6-8 week old) were injected subcutaneously with 1×10^6^ U87 cells in the left flanks on day 0 and randomized to 2 groups (n = 8 per group). Mice received 8×10^5^ PFU MV-Edm via tail vein injection every other day from day 10 to 18 and day 25 to 39. Total dose of MV-Edm for each mouse was 1×10^7^ PFU. Untreated mice were used as negative controls. Tumors were measured every 3 days, and tumor volume was calculated as length × width^2^/2.

### *In vivo* treatment by combining DCA with low-dose MV-Edm

Male Balb/c nude mice (6-8 week old) were injected subcutaneously with 1×10^6^ U87 cells in the left flanks on day 0 and randomized to 4 groups (5 to 6 mice per group). On day 6, two groups of mice were provided drinking water containing DCA (70 mg/L) until the end of the experiment. Starting 15 days after tumor inoculation, two groups of mice treated with DCA or untreated, were injected intravenously with 4×10^5^ PFU MV-Edm every 3 days from day 15 to 27 and day 36 to 42. Total dose of MV-Edm for each mouse was 3.2 × 10^6^ PFU. Tumors were measured every 3 days, and tumor volume was calculated as length × width^2^/2. Mice exhibiting moribund behavior were euthanized. All animal work was approved by the Animal Care Committee of Nanjing University in accordance with Institutional Animal Care and Use Committee guidelines.

### Statistical analyses

Student *t* test was used for all *in vitro* statistical analyses. The Mann–Whitney U-test was used for two-group luciferase activity comparison. Statistical analysis of tumor volume among the groups was done using repeated measures ANOVA. P < 0.05 was considered significant.
